# Composite Vulnerabilities and Hybrid Threats for Smart Sensors and Field Busses in Building Automation: A Review

**DOI:** 10.3390/s25175218

**Published:** 2025-08-22

**Authors:** Michael Gerhalter, Keshav Dahal

**Affiliations:** Artificial Intelligence, Virtual Communication and Network (AVCN) Research Institute, University of the West of Scotland (UWS), High St, Paisley PA1 2BE, UK; b00404591@studentmail.uws.ac.uk

**Keywords:** intelligent control, digital twin, fault diagnosis, building management system (BMS), attack vector, risk appetite, Modbus, M-Bus, KNX, BACnet

## Abstract

In the IT sector, the relevance of looking at security from many different angles and the inclusion of different areas is already known and understood. This approach is much less pronounced in the area of cyber physical systems and not present at all in the area of building automation. Increasing interconnectivity, undefined responsibilities, connections between secured and unsecured areas, and a lack of understanding of security among decision-makers pose a particular threat. This systematic review demonstrates a paucity of literature addressing real-world scenarios, asymmetric/hybrid threats, or composite vulnerabilities. In particular, the attack surface is significantly increased by the deployment of smart sensors and actuators in unprotected areas. Furthermore, a range of additional hybrid threats are cited, with practical examples being provided that have hitherto gone unnoticed in the extant literature. It will be shown whether solutions are available in neighboring areas and whether these can be transferred to building automation to increase the security of the entire system. Consequently, subsequent studies can be developed to create more accurate behavioral models, enabling more rapid and effective analysis of potential attacks to building automation.

## 1. Introduction

Almost all modern buildings have building automation systems (BASs) [1]. Literature clearly indicates that building automation can be classified as a cyber physical system (CPS) [2,3]. In this context, building automation combines several broader areas like Smart Buildings (SBs) [4], Intelligent Buildings (IBs) [5], smart cities [6], enhanced living environments [7], Information and Communication Technology (ICT), Industrial Control Systems (ICSs), smart grid [8], Internet of Things (IoT) [9] and others. This shows the necessity of combining many different and independent systems into one integrated system.

Khan et al. [10] recognize the interaction and interconnectivity between the different systems in the CPS environment through the use of ICT. With regard to BASs, for example, Aghemo et al. [11] introduced the topic of the energy-saving potential in heating, cooling, and lighting through ICT-based automation systems. Also, the EU‘s Energy Performance of Buildings Directive (EPBD) [12] recognizes the strong backlog demand regarding the intelligence capability of a building and its underlying controls. In the context of smart devices and sensors, the requirement to connect different BASs is also supported by Kastner et al. [13]. They specifically refer to the increasing requirement for close cooperation between the different trades involved in building automation engineering. Continuing towards broader integration, Hammadi et al. [14] are clear that indoor guidance and assisting via smartphone will become a significant trend in modern buildings. They also explicitly point to the interaction of smartphones and building automation as the key to implementing such a concept. In the context of linking electrical power systems and BASs, Kiliccote et al. [15] make clear that smart grids are also on the agenda for smart buildings. ASHRAE [16] has also started work on corresponding standards for the connection between building automation and smart grid systems. In the broader context of the CPS, Zhukabayeva et al. [17] highlight the integration of networked sensors into cloud applications, while also underscoring the significant number of accompanying security concerns.

The examples given of the diverse interconnection of different systems go hand in hand with a broad and complex attack surface [2,17,18], which is reminiscent of the challenges of modern warfare, and thus brings into play issues such as asymmetric or hybrid threats or composite vulnerabilities. Hybrid threats refer to a wide range of hostile actions that combine multiple, often unconventional methods to achieve strategic objectives, usually by exploiting multiple vulnerabilities in one or more targets [19]. Composite vulnerabilities are those vulnerabilities that can occur through the totality or combination of connected systems [20]. The question arises as to whether such threats are also conceivable in the field of building automation.

The following contributions are made by this paper:

- A comprehensive review of field bus systems, protocols and standards used for data transport from sensors in building automation.
- An overview of practical examples of threats to building security that have not yet been covered in the literature, especially with regard to sensor technology in usually unprotected areas.
- A thorough analysis of whether literature from the field of warfare or composite vulnerabilities has been previously applied to the field of CPSs and specifically to BASs.
- An overview of the methods that can be employed to counteract the mentioned composite vulnerabilities and hybrid threats for the benefit of researchers and practitioners in the field.

The previous part explained the background and motivation for this systematic review paper. The Section 2 describes the necessary context and the Section 3 shows some practical examples. Section 4 describes the methodology and defines the scope, focus, and limitations. The Section 5 summarizes and synthesizes the results in categories and describes them in detail, particularly with regard to their potential applicability in building automation. The Section 6 summarizes the most important results and concludes the key findings, by highlighting aspects of security in building automation that have not yet been considered in the literature.

## 2. Building Automation and Its Communication Mechanisms

### 2.1. The Multi-Layered Communication in Building Automation

**Figure 1:**
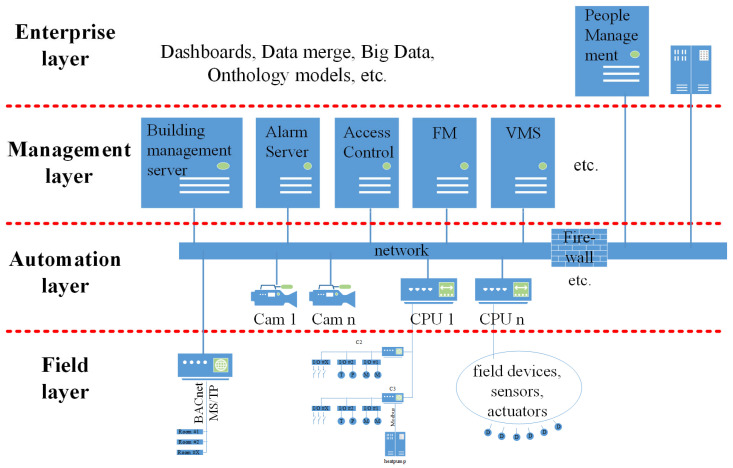
BAS automation layer.

**Figure 9:**
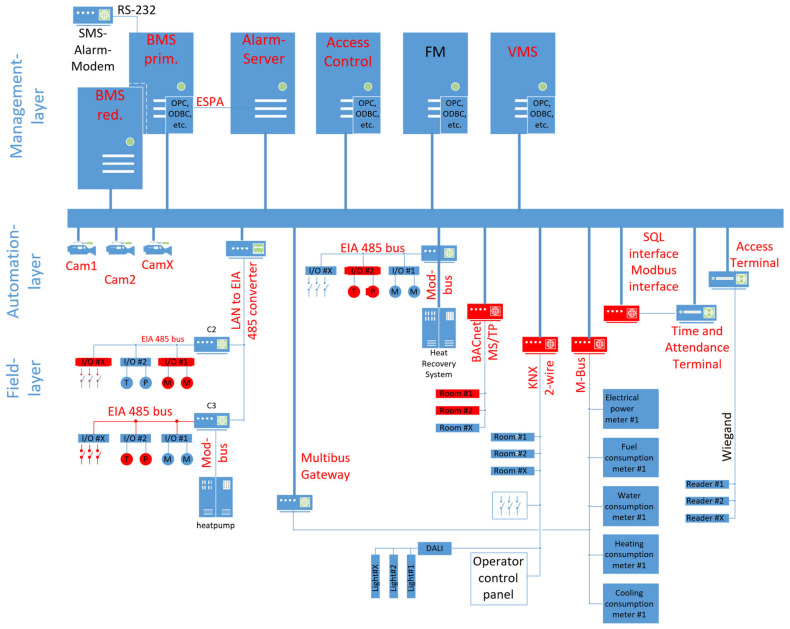
A practical example of devices and their connections between layers that are often unprotected.

Building automation systems are divided into layered communication, which is supported by several authors and widely used in the literature [21,22]. To better illustrate these layers with a picture, Figure 1 shows the typical four layers of a BAS and their underlying communication mechanisms. Figure 1 also demonstrates the various types, distribution, and number of devices utilized in building automation. The majority of these devices can be found at the field layer, with their distribution spanning the entirety of the building. For example, an outside air temperature sensor sits usually on the outside wall, occupancy sensors are located in rooms or corridors, and sensors for Air Handling Units (AHUs) are located at the AHU itself. Furthermore, a plethora of additional devices, including temperature and humidity sensors, valve actuators, fire alarms, access readers, cameras, and numerous others, have been developed for integration into these systems. The automation layer is constituted by the Direct Digital Controllers (DDCs), which are utilized for the purpose of automating the processes. These devices are typically installed in control cabinets, though they are also sometimes distributed throughout the building. The management layer, situated above this, is employed for the purpose of visualizing processes and automating higher-level processes. The workstations and servers used there are usually located in an office or a dedicated server room [21]. The enterprise layer is utilized for the processing of voluminous data sets and the establishment of connections with higher-level systems, including the organization’s IT infrastructure. Data exchange in horizontal communication is primarily used for process control and is characterized by smaller data volumes. Data exchange in vertical communication is primarily intended for the access by the management level and is typically characterized by higher data volumes, e.g., for historical data collection [13]. The threats posed by these connections are explained later in Section 5.4.3. and Figure 9.

### 2.2. Cross-Level and Cross-Trade Vulnerabilities

Sinopoli [23], Younus et al. [24], and Butzin et al. [25] note that building automation is a sum of many different trades such as access control, CCTV (Closed-Circuit Television), HVAC (Heating Ventilation and Air Conditioning), intrusion detection systems, fire alarm systems, etc., whose underlying BASs are interconnected in many ways via a variety of gateways or fieldbus systems. Macaulay [26] notes that, in the context of IoT, threats and vulnerabilities especially occur due to the interconnectivity of many different devices, and the emerging of gateways and cloud solutions even increases the attack surface of such systems [27]. Pointing to IoT and large BASs, Brooks et al. [28] note that the emerging interconnectivity of BASs and enterprise systems poses a new and still far too little considered threat to organizational security, while their reference to the security of the organization is that the connection between the technical and organizational LAN (Local Area Network) also increases the risk of attacks. In the majority of cases, the supply and implementation of these disparate systems is undertaken by different manufacturers. These manufacturers frequently engage in competition with one another, exhibiting a limited inclination to collaborate, and demonstrating a preference for the provision and implementation of proprietary solutions, as opposed to those that are security oriented. This observation is also noted by Shwartz et al. [29] in relation to the IoT. This paper examines literature pertaining to vulnerabilities at the various BAS layers and the different trades. It specifically analyzes literature that already deals with this topic in related areas, with a view to highlighting the associated composite and hybrid vulnerabilities.

## 3. Practical Examples of Hybrid and Composite Vulnerabilities

The subsequent practical illustrations are designed to fulfill the following objectives:

- Help to understand the concept and criticality of composite vulnerabilities and hybrid threats to building automation.
- Provision of illustrative examples that have not been previously addressed in the extant literature.
- Documentation of real-world scenarios in order to improve data management and strengthen real-time capabilities of fault detection, as these have been given very little consideration in the literature to date [30].

The examples cited are solutions that have also been implemented in practice. During the implementation phase, the risks cited were discussed, and it was determined that there is a paucity of literature examining composite vulnerabilities and hybrid threats in building automation.

### 3.1. Example 1, Composite Vulnerabilities

**Figure 2:**
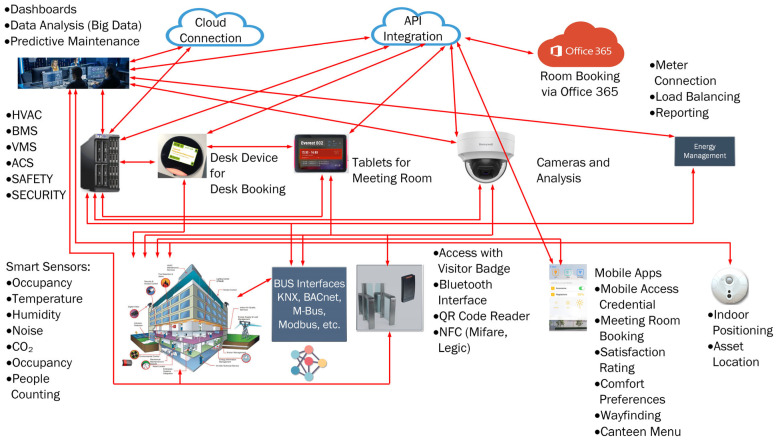
Practical example of interconnectivity in building automation.

A visitor to a building is sent a QR code to their mobile phone after a meeting has been booked via a Microsoft Outlook calendar appointment object. They also receive a floor plan to make it easier to find their way to the relevant meeting room. The visitor then enters the building by presenting the QR code to the access card reader at the entrance door. This event data is stored in the Access Control System (ACS) and recorded by the Video Management System (VMS). If it is an employee, the data is also sent to the human resources payroll system to recognize the employee’s presence and set their daily attendance account to ‘active’. In addition, the visitor’s path to the office is automatically lit when it is dark and the office space is heated or cooled to the desired temperature. The blinds are also opened or closed depending on the weather conditions. Furthermore, the presence detectors recognize whether people are still in the room and the media control system activates the appropriate lighting scenarios for a presentation. In other words, an ACS is connected to the Time and Attendance (T&A) system, to the payroll system, to the lighting system, to the HVAC, to the VMS, to the media control panel, to the electrical power supply system, and in some way to the mobile phone of an external person. Figure 2 illustrates this scenario.

Figure 2 also shows a strong fusion between IT and OT (Operational Technology) and a correspondingly strong networking between the various automation systems, which is also currently the focus of the literature [17,31]. In addition to the broad attack surface [2], such scenarios offer the potential for attacks on one system to affect multiple, other connected systems and support the spread of malware.

### 3.2. Example 2, Composite Vulnerabilities

In an airport there is a car rental for electric cars. The power supply of the loading stations does not provide enough power capacity to load all vehicles in parallel. To ensure that the rented vehicle is fully charged at the time of handover, a charging schedule is created. This schedule defines the time and corresponding State of Charge (SOC) of the battery. This data is transferred to an SQL (Structured Query Language) database, which is then connected to a controller via an ODBC (Open Database Connectivity) connection. The connected controller is then also used to disconnect other large loads when larger amounts of power are needed for rapid charging. Thus, the controller is connected to many AHUs (via FOXnet protocol), an electric heater for defrosting a ramp (via ModBus), to the refrigeration compressor network of the entire refrigeration supply (via BACnet), and to the other vehicle loading stations (via Modbus). This common and widespread use of unsecured connections supports potential cyberattacks and represents a large, poorly secured attack surface. For example, an attack could be carried out via the BACnet weather station, which is often poorly secured outdoors, or via BACnet room control units. Furthermore, an attack on a single system can have significant consequences for other systems. For example, an attack on the controller, which is located in the control cabinet of the ventilation system and is therefore more easily accessible, can also result in the de-icing of the access ramp being disabled, which also poses a significant risk of accidents.

### 3.3. Example 3, Composite Vulnerabilities

Following Nge et al. [8], who had already identified the direct connection between the smart grid, the energy management system, and the HVAC system as a potential threat, such scenarios are also being implemented in practice. For example, energy meters are connected directly to the controller via unsecured protocols (M-Bus, Modbus, BACnet, etc.), which are then often directly connected to enterprise dashboards or cloud solutions. On the one hand, this makes cyberattacks on unsecured protocols easier, and on the other hand, the connections to cloud solutions and enterprise networks make it much easier to spread malware.

### 3.4. Example 4, Hybrid Threats

In buildings, there are usually one or two large air handling units supplying the whole building with fresh air (such as classrooms, event rooms, offices, patient rooms, exhibition rooms, etc.). These air handling units are often equipped with an outside air intake at floor level, which represents a major vulnerability. This allows potentially harmful or lethal gases to be placed near the intake of the ventilation system, causing significant damage and even life-threatening situations without much effort. While this example does not refer to sensors in unprotected areas or unsecured data transmission, it clearly demonstrates the vulnerability of ventilation systems to hybrid attacks. The deployment of sensors within the intake tract, capable of detecting noxious gases, has the potential to serve as a partial countermeasure against such attacks. However, there is a high risk that the harmful gases will be detected too late or not at all, as the sensors cannot respond to all dangerous gases. Consequently, the most rational approach would be to install the intake ducts in inaccessible areas, though this frequently results in elevated installation costs.

## 4. Methodology

The overall aim was to find literature that can help to improve the security of buildings with building automation systems. Methods from other areas were analyzed and then projected onto the security of buildings in general. The security of buildings in this work is to be considered holistically, in order to be able to evaluate as many vulnerabilities as possible.

### 4.1. Design of the Literature Review

**Figure 3:**
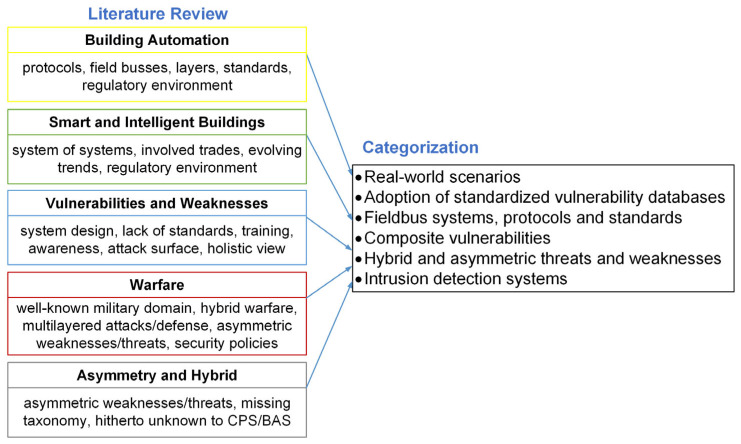
Areas included in the literature review and their categorization.

Firstly, the current state of the art in building automation was determined with the aim of demonstrating the implementation or non-implementation of various security mechanisms. This analysis then served as the basis for the applicability of the further topics of investigation. Figure 3 shows the areas reviewed in literature and their categorization into real-world scenarios, adoption of standardized vulnerability databases, fieldbus systems, protocols and standards, composite vulnerabilities, hybrid and asymmetric threats and weaknesses, and intrusion detection systems. The authors created the categorization in an iterative process based on the literature examined, which ultimately resulted in these categories. Different and contradictory approaches are demonstrated in the following sections, as well as their compatibility with the analyzed domains.

### 4.2. Review Method and Selection Process

**Figure 4:**
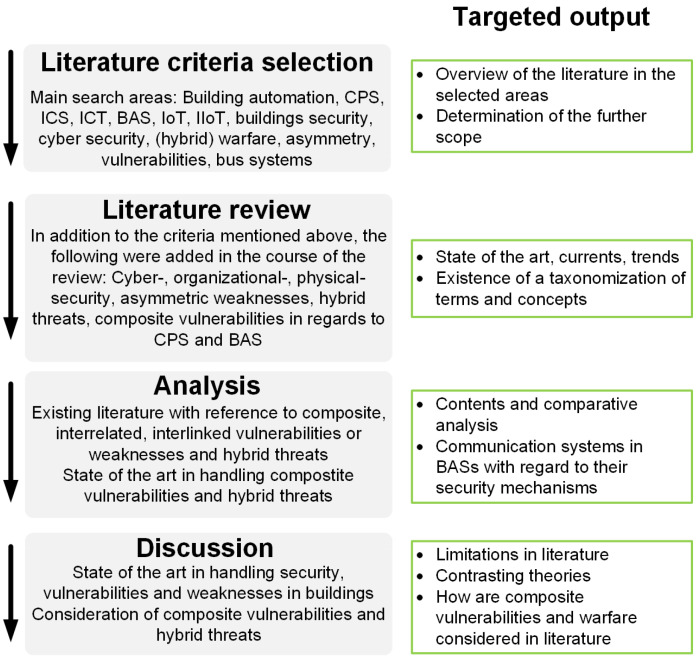
Structure and objectives of the review.

The search areas were intentionally chosen broadly to cover most possible areas which can contain any information about composite, interrelated, interlinked, hybrid, and asymmetric vulnerabilities or weaknesses. The articles were first selected on the basis of the title; if inclusion of the title was selected, further selection was made on the basis of the abstract. The articles were analyzed independently and primarily by the author of this review. In instances of uncertainty, the second author was approached for consultation. No automation tools were used in the review process. Figure 4 shows the methodological process and the desired result of each sub-step.

### 4.3. Inclusion and Exclusion Criteria

In order to keep the search scope as broad as possible, the following types of literature were analyzed: books, conference papers, government documents, journal articles, legal rules or regulations, reports, standards, electronic articles, online databases, newspaper articles, press releases, and webpages. No inclusion or exclusion criteria were defined for authors, the cite score, or geographical affiliation. Because of the longevity of BASs [32], no time restriction was set for the initial searches in order to obtain an overview of the relevant areas. However, if more recent literature on the same topic was available, the more recent literature was favored.

#### 4.3.1. Further Inclusion Criteria

- Literature addressing composite, interrelated, or interlinked vulnerabilities in the context of CPS, ICT, ICS, and BAS.
- Literature addressing hybrid or asymmetric threats or vulnerabilities in the context of CPS, ICT, ICS, and BAS.
- Literature related to hybrid or asymmetric warfare in connection with CPS, ICT, ICS, and BAS.

#### 4.3.2. Further Exclusion Criteria

- Literature regarding safety, natural hazards, or events such as floods, storms, earthquakes, avalanches, and similar.
- Literature related to terrorist attacks was excluded.
  ○ Unless it explicitly fell under the above-mentioned categories and was relevant in the context of the article being analyzed.
- Literature in the context of data privacy or GDPR (General Data Protection Regulation).
- Non-indexed government documents, journal articles, legal rules or regulations, reports, standards and preprints to assure accessibility and academic rigor.
- Only reports written in English or German were analyzed. Reports in all other languages were excluded.

### 4.4. Search Procedure

The platform used in the initial searches was Google Scholar, as it also performs a search in established research platforms and offers broader coverage across different disciplines [33]. For further, more detailed research on the specific topics, a separate search was then carried out via the following sources: eBook Collection (EBSCOhost), Science Direct, IEEE Xplore, Scopus, and Web of Science. A total of 131 search strings were applied, which were then used in the various search queries on the respective topics. The initial search terms were taken from relevant literature on the subject of security in building automation, smart buildings, and intelligent buildings [22,34,35,36,37].

### 4.5. Data Extraction and Presentation

Due to the diversity of the areas analyzed, the literature extracted was summarized in six categories. This was intended to provide a better overview and summarize the most important topics and current trends in the literature.

### 4.6. Quality Declaration

**Figure 5:**
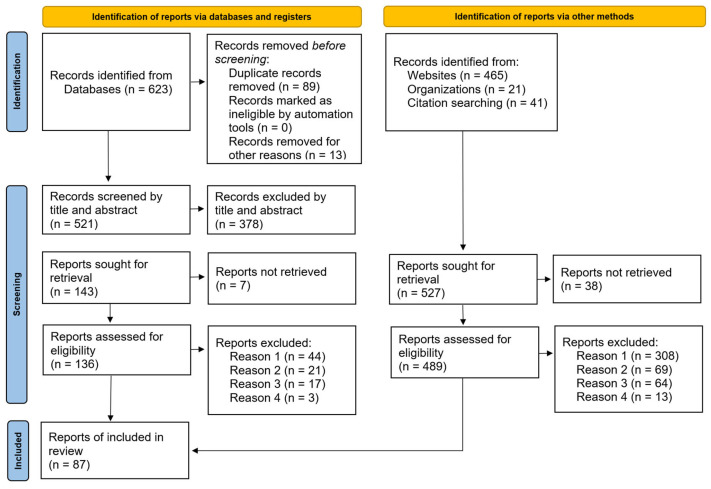
Flow diagram on the basis of PRISMA [38].

The review was mostly conducted following the PRISMA framework guidelines and the quality criteria and checklists of the Prisma framework [38]. The corresponding flow diagram is shown in Figure 5. As per the partially semi-structured approach, there is a possibility that the results may be distorted, as not all areas of literature on the topic of security in buildings with building automation were possibly found. This bias was counteracted by analyzing all the reports examined for evidence of other threats or vulnerabilities related to buildings.

A total of 1150 documents were identified for examination, of which 87 are referenced in this review. Description of the reasons for exclusion are listed in Figure 5:

- Reason 1: The inclusion criteria have not been fully met.
- Reason 2: Exclusion criterion: Literature regarding safety, natural hazards, or events such as floods, storms, earthquakes, avalanches, and similar.
- Reason 3: Exclusion criterion: Literature in the context of data privacy or GDPR.
- Reason 4: Other reasons for exclusion according to the exclusion criteria.

## 5. Results of the Literature Review

Table 1 shows the summary of the extracted literature and its categorization. The following sections provide more detail on the respective topics.

### 5.1. Occurrences of Real-World Scenarios in the Literature

Articles in the CPS area on threat classification or vulnerabilities related to asymmetry are rare, appearing in only 8 out of 29 articles examined. This is essentially also supported by [39], who suggests that it is important to taxonomize asymmetries in order to better understand how to deal with the corresponding vulnerabilities. Some approaches on new attack vectors for BASs have been made [40]. In the area of IoT, further contributions deal with the adaptation of existing, standardized databases [41,42] that can be followed up and adopted in regards to BASs.

**Figure 6:**
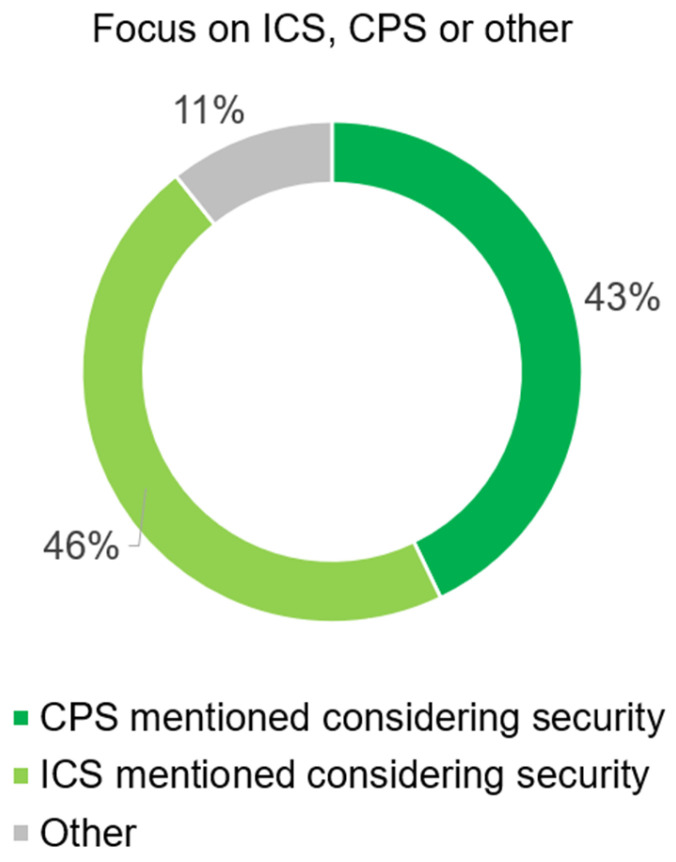
Distribution of literature with focus on security in the areas of ICS and CPS.

**Figure 7:**
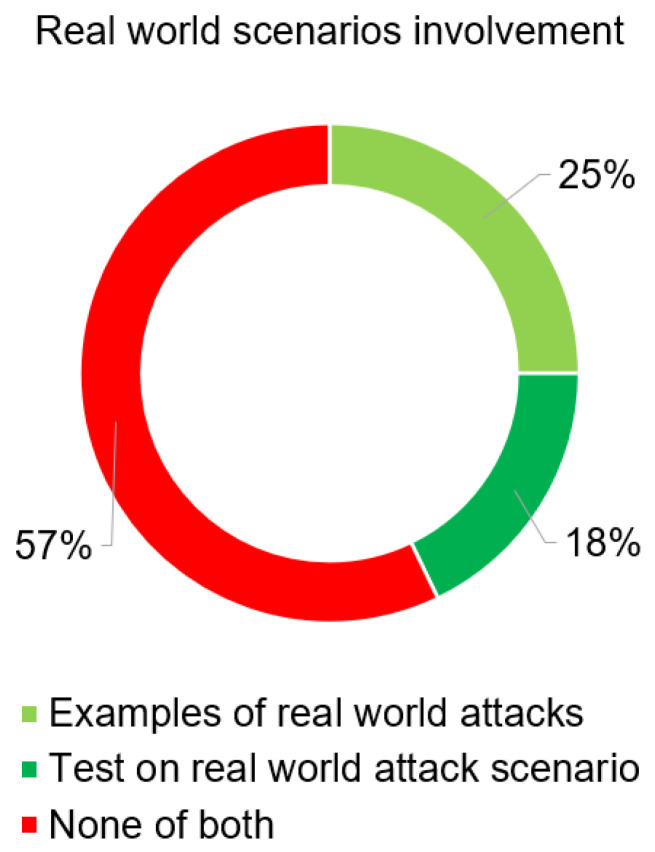
Distribution of literature with focus on real-world scenarios involvement.

The distribution of the reviewed literature in the areas of ICS, ICT, and CPS is almost equally distributed, as shown in Figure 6. In terms of practical applicability, the distribution of real-world scenarios is interesting, as shown in Figure 7. The sum of real-world examples and real-world tests scenarios occurs in less than 50% of the literature examined, thus the theoretical treatment of vulnerabilities, weaknesses, and threat scenarios predominates, which is essentially confirmed by [31].

### 5.2. Adoption of Standardized Vulnerability Databases for CPS and BAS

Looking at the Common Vulnerability Scoring System CVSS [43] or the National Vulnerability Database (NVD) [44], a problem with the various vulnerabilities and their classification is that the information content is sometimes difficult to understand for CPS operators. Often the vulnerabilities are described rather vaguely as information break, distorted input value, channel weakness or similar, which sometimes has little practical significance, allows little conclusion about the effect or the source, or just misses the context to the application [28]. The origin of these designations usually comes from computer technology and poses great challenges for CPS or BAS operators in terms of understanding the statements, as these statements are too focused on the area of network technology or general IT [45]. Without appropriately trained personnel or corresponding specialist departments, such vulnerability reports are therefore mostly useless for CPS and BAS owners, or their informative value can usually not be interpreted appropriately and is even less implemented in countermeasures. In their current form, these databases are therefore rather unsuitable for use in the BAS area.

### 5.3. Fieldbus Systems, Protocols and Standards in BAS with Regard to Security

#### 5.3.1. Analysis of Fieldbus Systems Used in Relation to Their Security

**Figure 8:**
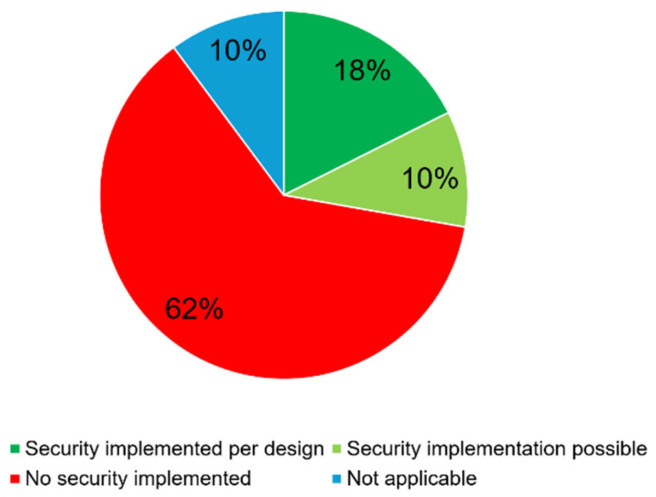
Protocols, standards, and field bus systems with or without security implementation.

In the context of ICT, Supervisory Control and Data Acquisition (SCADA), and Distributed Control System (DCS), the National Institute of Standards and Technology (NIST) created an overview of all the corresponding threats and vulnerabilities and provides guidelines to mitigate the associated risks [46]. There is a comprehensive survey of Industrial Internet of Things (IIoT) protocols [42], which is also applicable to some parts of building automation. However, their focus was on IIoT and thus many protocols used in building automation were not investigated. This gap was closed by analyzing 108 protocols and fieldbus systems used in BAS and their implementation of security mechanisms. Figure 8 shows that only 19 of the 108 protocols and fieldbus systems analyzed have implemented security by design and 11 of them have the option of selecting a security mechanism. The vast majority of fieldbus systems, protocols and standards used in building automation can be classified as insecure, at 62%. It should be noted that 42 of the examined protocols are proprietary systems. In these systems, the data transmission protocol is the intellectual property of the manufacturer and is therefore not disclosed. While this increases security to some extent, it does not match the security of a transmission protocol with a built-in encryption mechanism. This suggests that secure systems do exist and can be implemented in practice, but the majority of currently installed systems require significant improvement in terms of fieldbus security.

Furthermore, the potential penetration path via discovery tools has not yet been considered in the literature. Table 2 shows that 15 of the 108 bus systems analyzed enable automatic detection of all bus devices and usually also their entire objects, including the control and regulation parameters. This means that, for BACnet as an example, the ‘Who-Has’ service can be used to determine where certain devices or objects are located without having to know the exact addresses of all devices in the network. Together with the ‘Who-Is’ command, the ‘Who-Has’ service helps to determine the network addresses and object IDs of objects that are located in other BACnet devices. Most protocols also utilize the option of transporting their messages via TCP/IP packets. In practice, this leads to cases where smart sensors in unprotected areas are connected via two-wire bus systems and then connected directly to the OT via gateways using TCP/IP. In addition, they are often also connected directly to the organization’s IT network, as described in Section 3.1, and shown in Figure 2.

#### 5.3.2. Security Strategies at the Field- and Automation Layers

In principle, the implementation of security mechanisms in fieldbus systems already significantly increases security. For example, implementing data encryption would make it significantly more difficult or even impossible to infiltrate malicious devices or eavesdrop [47,48,49]. This is already possible with BACnet Secure or KNX Secure, for example, but is rarely implemented in existing installations. Furthermore, relevant stakeholders still lack knowledge of how to implement BACnet Secure or KNX Secure. In access control systems, for example, switching to mutual authentication between the card and the reader, or encrypted data transmission for read/write access, would also increase security. The drawback is that such implementations are expensive, so they are usually not implemented in existing installations. Considering the longevity of BASs [32], security mechanisms at the field and automation layers are not usually implemented or added to existing systems, only being implemented when the systems are replaced.

### 5.4. Composite Vulnerabilities in Relation to CPS, ICT, ICS, IoT, and BAS

#### 5.4.1. Literature Around Composite Vulnerabilities in the BAS, CPS, ICT, and ICS Areas

Considering BAS as a ‘system of systems’ with the many connections within the layers and also among each other and between the various trades, new vulnerabilities can arise that are ignored or do not occur in the individual consideration. This perspective was taken up by Ciholas et al. [20] in the higher-level context of CPSs. The resulting system vulnerabilities are referred to as ‘composite vulnerabilities’. It is also noted that organizations such as NIST (National Institute of Standards and Technology), CPNI (Centre for Protection of National Infrastructure), and similar entities have not yet addressed this subject, or have only focused on individual vulnerabilities in their publications, or have used an IDS (Intrusion Detection System) to focus on attacks that are already in progress. New vulnerabilities that can result from the aggregation of different systems or individual vulnerabilities were named as ‘emergent vulnerabilities’ [50]. They try to counteract this complexity of systems from different points of view, for example, by considering the adversary goals, existing cyber and threat databases, or attack-centric analysis. In the area of information systems, Qu et al. [51] mention that there is no way to objectively measure composite vulnerabilities. Besides their general observation that there are currently no established systems for measuring interrelated vulnerabilities in information systems, they point out that there are already established methods for measuring individual, independent vulnerabilities such as the CVSS or the NVD. However, in their specific example, they found that CVSS is not able to measure composite vulnerabilities.

Also in the context of composite vulnerabilities, but not mentioning the term explicitly, refs. [32,35] mention the use of smart sensors and actuators that are connected to the automation layer via a bus system like KNX, BACnet, LON, etc. They point out that the possibility of local access to these bus systems leads to considerable vulnerabilities at the field layer, especially since smart sensors and actuators are often installed in unsecured areas [52,53]. This observation is particularly interesting in conjunction with the study by Ciholas et al. [52], who point to the penetration of threats between the three layers in a BAS during attacks. This can mean that attacks carried out in the often unsecured field layer can lead to the distribution of malware throughout the BAS network. This is in contrast to Brooks et al. [28], who state that local access to field devices only creates local vulnerabilities limited to small parts of the BAS.

#### 5.4.2. Literature Around Composite Vulnerabilities in the IoT Area

In the field of IoT, the networking of many different devices is a fundamental issue and has already been addressed in the literature. For example, a reference is made to the vulnerabilities of conflicting rules between different devices. Mutual influence, vulnerabilities arising from interaction between devices, and inter-rule vulnerabilities are cited as potential threats [54,55]. However, when the BAS is compared with IoT, these vulnerabilities are relatively minor in the context of building automation, since the control rules for automation applications are typically developed by qualified experts. In addition, the control requirements are thought through in advance by planners or end customers and documented accordingly. However, the vulnerabilities remain due to the networking of different devices. This includes the potentially possible impact of an attack on all devices in a control network, which essentially corresponds to the principle of composite vulnerabilities. The issue of diversity among numerous manufacturers, whose hardware and sensor technology are frequently found to be insecure, is also recognized in the context of the IoT, and has already been addressed in the extant literature. Specifically, ref. [29] describes how it is often assumed that data received from other manufacturers is secure, even though it is usually connected via unsecured protocols, as demonstrated in Section 5.3. This predicament gives rise to the issue that hardware-based attacks on CPSs provide an opportunity for attackers to impersonate authenticated and privileged users and also suggests the potential for causing damage to the system without leaving many traces. In the context of building automation, this poses a risk that untested manufacturers or suppliers may be integrated into larger systems without the presence of appropriate system integrators who could potentially monitor these processes [28].

In relation to the utilization of contemporary technologies, Cimino et al. [55] adopt a methodology that employs LLMs (Large Language Models) to identify and counteract conflicting or mutually influential control rules. No literature was found in the field of building automation that uses LLMs or other AI (artificial intelligence) models to increase security, with the exception of the utilization of IDS to detect anomalies, further details of which can be found in Section 5.6. Presently, AI models are predominantly employed in the context of BASs for the purposes of predictive maintenance and the optimization of control strategies, for example [56].

#### 5.4.3. Strategies to Mitigate Composite Vulnerabilities in BASs

Composite vulnerabilities arise from the connection of different systems. Thus, data from a falsified or influenced source can cause damage to another target system. Figure 9 shows a detailed diagram illustrating the diverse data connections between the various devices that are marked in red. This demonstrates that the multiple connections between devices at different layers could make it potentially quite easy to spread malware. Furthermore, Figure 9 illustrates the increased attack surface, caused by the numerous bus systems utilized in building automation. Given these unprotected data sources, introducing trust relationships would enable source data to be integrated into the overall system in a verified manner. One option is to use certificates, which can be used to secure communication from the field layer through the automation layer to the management or enterprise layer. For example, this could be achieved using BACnet/SC, which enables secure communication at the field layer [48,49]. But this would only apply to the HVAC sector. Another option would be to divide the various providers into many, very small network segments [24,57]. This usually involves significant administrative effort, however, and is limited to the IP network level. The division does not extend to the field layer. In order to better protect the diversity of systems used in building automation as a whole, a position should be created that deals with all systems, their integration, and their data connections. One such function would be the system integrator [48], who monitors all systems and not only checks and approves security-related functions, but also considers conflicting rules [54,55]. It should also be noted that in high-security applications such as data security centers or military facilities, this function is sometimes performed by security planners. But such planners usually consider many aspects of building automation, such as heating systems, to be irrelevant to security and thus ignore potential threats, which are discussed separately in Section 5.5.

### 5.5. Hybrid and Asymmetric Warfare Related to CPS, ICT, ICS, and BAS

#### 5.5.1. Literature Related to Hybrid and Asymmetric Warfare in Connection with CPS, ICT, ICS, and BAS

Although from different angles, relevant asymmetric challenges have been extensively studied by only the defense [58,59] and cyber-security research communities [60]. Asymmetric tactics are an important part of the history of warfare. For example, Miles et al. [61] emphasize the need to exploit the opponent’s strengths and weaknesses and use them accordingly. It has been established that nations, organizations, and individuals have either discovered opportunities to use ICT to benefit from asymmetric weaknesses, or, conversely, are threatened by asymmetric weaknesses [62]. The North Atlantic Treaty Organization (NATO) defines hybrid threats rather generally and also includes all asymmetric conflict scenarios, low-intensity threats, cyber-terrorism, organized cyber-crime, and others [19]. Due to relatively recent and rapid developments, the building automation community has yet to address this area.

By mentioning the increasing integration level of automation systems, Mahmoud et al. [18] point out that insecurities of the physical layer are intertwined with the design of the application controller and both must be considered accordingly in the design of the security policy for the entire system. Their reference to the necessity of looking at the whole system was also investigated by [63] in relation to asymmetric warfare. They found that aggressors who have a massive resource disadvantage will utilize asymmetric techniques to a maximum, whereas their definition of asymmetric techniques is that of achieving the best ‘cost-benefit ratio’. Thus, attackers only have to look for the weakest point in the entire system, which often leads to even the most experienced defenders not being able to correctly assess the situation in the attack scenario. In the context of CPSs, this topic was taken up by Gupta et al. [64]. Their work is based on the analysis of a Denial of Service (DOS) attack on the CPS where they try to formulate the behavior of attacker and defender as well as possible mathematically in order to be able to carry out a corresponding simulation. They also showed that the scientific community has become increasingly interested in the diversity of securing CPSs in recent years.

#### 5.5.2. Literature Related to Asymmetrical or Hybrid Weaknesses in Connection with CPS, ICT, ICS, and BAS

In recent years, the term ‘asymmetrical-weaknesses’ threats or vulnerabilities’, also called ‘hybrid threats’ in the context of CPS and ICT, came into play [65,66]. Asymmetry is also cited in relation to the information asymmetry between the attacker and the defender, mostly in reports aiming at general IT security issues [67,68]. A start was made on taxonomizing the concept of asymmetry in a literature review, not in relation to CPS or ICT, but with a focus on security and privacy in networks [69]. With regard to CPS, there is already a good approach to identifying system weaknesses using STRIDE (Spoofing, Tampering, Repudiation, Information Disclosure, Denial of Service, Elevation of Privilege) and evaluating the associated risk using DREAD (Damage, Reproducibility, Exploitability, Affected Users, Discoverability) [70]. Potential system interdependencies and asymmetric threats were also taken into consideration. However, the approach is theoretical and no real-world scenarios are explicitly cited or analyzed. Furthermore, no study was found that linked the issue of asymmetric or hybrid threats and BASs.

Considering the BAS and its limited resources in field devices like memory, computing capacity, power restrictions, etc. [21], it is currently not possible to implement sophisticated, up-to-date security mechanisms [47]. This brings in another factor of asymmetry, namely the difference between the different layers in building automation, which can also be considered as asymmetry [65]. This is broadly in line with [52,53,71], who note that field devices are often located in unsecured areas, leading to further asymmetry in terms of the attack surface of a BAS. This also means that the sum of possible, physical entry points for BAS devices in unsecured areas is higher than for devices in secured areas. Thus, possible perpetrators have several possible points of attack through which the perpetrator can access the BAS behind the device, or even multiple systems if the BAS is connected to them [24,25]. In addition, modern fieldbus systems are usually also available at the IP level [72], which transfers the vulnerabilities from the field layer up to the IP layer, or even the enterprise network [73], if this is not appropriately secured by firewalls or gateways.

#### 5.5.3. Strategies to Mitigate Hybrid or Asymmetrical Weaknesses in BASs

From warfare [59] and CPS [64], it is known that assessing and defending against hybrid threats is a complex process and usually involves many different areas. The literature on the CPS and neighboring areas is still being developed. The Common Weakness Enumeration [36], NIST [44], and ENISA [74] have started working with Threat Modelling Methods (TMMs), which only cover hybrid threat scenarios to a limited extent. Since these only partially cover hybrid threats or are not directly or easily applicable to CPS, ref. [75] attempted to apply the topic of hybrid threats to the area of ICS using STRIDE [10], the CVSS framework [36], and other methods. With reference to BASs specifically, possible methods for mitigating hybrid threats would be based on the examples cited in the above literature. For example, access to unsecured fieldbuses could be prevented more effectively by consistently locking all web servers, gateways, and connection terminals, both inside and outside the building. However, building automation also includes many smart sensors, such as room temperature sensors, light switches, bus couplers, weather stations, etc. Such devices are very difficult to protect against unauthorized access. These would then have to be separated, as also mentioned in Section 5.4.3., ideally by very small, structured networks in order to minimize the damage in case of an attack or to limit it to smaller system areas. In contrast, considering the attack on the outside air intake system cited in example 4, locating the intake ducts on the roof or in inaccessible areas would be a possible strategy to minimize such a risk. In such cases, installing sensors in the air intake tract that detect harmful or lethal gases would be of little use. For detection to occur, the gas would already need to have entered the ventilation unit, which could potentially cause damage even if the ventilation system were immediately shut down and the outside air dampers closed. However, such points would need to be considered during the system planning phase as part of a ‘security by design’ approach. This is because changes to existing systems are usually too costly to implement. As a simple immediate measure, facility management staff and other stakeholders who influence building security could be trained on such threats in order to create appropriate awareness. An expert survey is planned to gain a better overview of hybrid threats to building automation. This survey will analyze the security practices of experts with significant influence over building automation security, with the aim of developing more appropriate measures to improve security.

### 5.6. Intrusion Detection Systems in CPS

**Figure 10:**
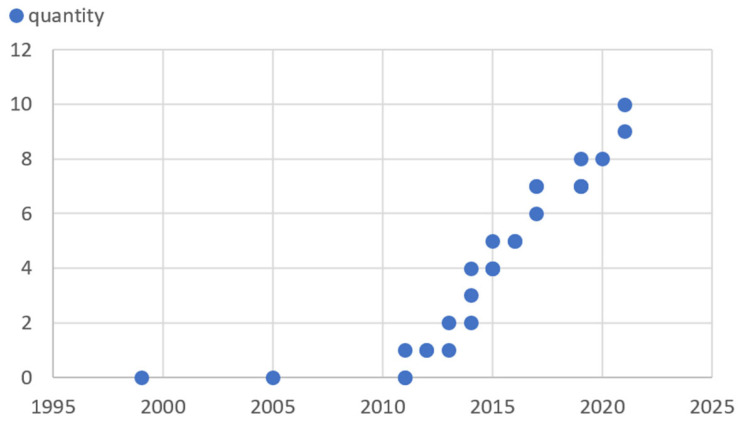
Literature around intrusion detection systems in CPS.

It is also apparent that there is a clear trend towards the use of behavioral models for cyber-physical processes to detect intruders for cyber-attacks. Figure 10 shows that beginning from 2011, the idea of for monitoring the system behavior of the entire physical process has already been thought of for more than ten years [76], whereas in the first reports, it was still assumed that the attackers have access to the configuration system, which might not cover too many attack scenarios. Shortly afterwards, around 2013, references had already been made to industry-standard machine learners for attack detection in ICT applications, which thus maps a function of an IDS [77].

Intrusion detection systems are usually classified into three types: signature-based, which detect based on documented behavior, anomaly-based, which detect based on machine learning including history data analyses, and hybrid, which is a combination of signature- and anomaly-based systems [78]. Most of the current IDSs which have their origin in the IT sector focus on the behavior of network traffic, which is not sufficient for a reliable detection of all attack vectors in a CPS [79,80]. This is also supported by Zhang et al. [78], who add that there are still too few studies on the subject of cyber security in relation to process data. Considering the building automation area, the literature review has shown that there is no literature on the topic of behavioral analysis for anomaly detection, threat prevention, or intrusion detection. Implementing behavioral model analyses for use as an IDS has a lot of advantages. Especially in CPSs, the thought of implementing security comes as an afterthought. This is often due to the fact that security requirements are mutually exclusive to functional requirements [81]. In addition, cost constraints often preclude the implementation of security by design in the early stages of CPS planning.

### 5.7. Discussion on the Utilization of Behavioral Models in BASs

If security was not implemented by design, a behavior model-based IDS could be used as a later workaround. The use of a digital twin in BASs as an IDS would be an interesting research work, as the digital twin delivers situational awareness of the whole CPS or BAS. If there is already a digital twin available in a BAS, e.g., for energy consumption modelling or predictive maintenance, the same model can be used as a base for an IDS. Dedicated literature in this topic was not found in this review. Table 3 provides an overview of existing research into behavioral models in the context of BASs.

With regard to security monitoring in BASs, for example, Graveto et al. [22] suggest using dedicated devices in addition to the control equipment already implemented, which would then detect anomalies and potential attacks. This is in contrast to most proposed solutions in IDS, which are based on behavioral model analysis and would also not be feasible to implement in BASs, as limited memory, computing capacity, and power restrictions of the devices at the field and automation layers would not be sufficient for this purpose [21,47]. In addition to the limitations mentioned above, there are also other challenges for behavioral models in building automation, for example, the limited bandwidth of fieldbus systems, their high latency, and their highly variable network traffic due to many loosely coupled devices. This is also partially confirmed by Jefrey et al. [31], whereby they point out that further research is necessary in the area of more complex learning models in large and heterogeneous systems in order to achieve better recognition accuracy.

### 5.8. Security Mechanisms from Other Areas

The field of CPS is broad, and the security mechanisms discussed in the literature are extensive. To cover as many potential security enhancements as possible, literature from the fields of smartphones, automotive technology, and sensor security was also examined at random. The aim was to investigate their applicability to building automation and to identify possible new areas of research. In the smartphone sector, for example, sensors are used for facial or iris recognition and fingerprint scanning. Many applications also use two-factor or multi-factor authentication. Other methods are also being worked on, such as the introduction of audio factors as a further authentication factor [85,86]. Currently, such methods are difficult to implement in building automation systems due to limitations in computing and memory capacity. Additionally, the required sensor technology would incur significant costs [21]. Another interesting approach is that of side-channel attacks, which pose a threat to authentication systems that use fingerprint scanners or facial recognition technology. These threats are already documented in the literature on biometric authentication [87]. The same applies to the field of sensor security in automotive technology. At the present time, physical attacks on sensors, for example, those employed in the context of license plate recognition, are a subject that is being addressed in the extant literature. Of particular concern are attacks that utilize fluorescent materials, ultraviolet light, or laser light, as these have the potential to significantly disrupt detection through the use of rudimentary methods. However, the relevance of these attacks to building automation is negligible, and their projection onto such systems is difficult. However, given the variety of systems used in building automation, biometric authentication plays a rather minor role. Nonetheless, when acquiring such systems, it is imperative to pay particular attention to certified products in order to minimize the resulting hybrid threats.

## 6. Discussion and Conclusions

Asymmetric attacks and hybrid warfare are well understood in the military domain because there have been studies for decades. In comparison, the IT revolution is still very young and ongoing. Therefore, from a scientific point of view, the taxonomy of cyber vulnerabilities is still immature and the process of categorization is not yet complete. In addition to the security vulnerabilities in the cyber domain, buildings also have potential vulnerabilities in the physical domain that are intertwined with those in the cyber domain. Furthermore, when considering an integrated system such as the BAS, it is imperative to acknowledge the significance of the social and organizational perspectives that it encompasses.

This systematic review has shown that existing literature focuses predominantly on cyber, physical, or organizational vulnerabilities in isolation. The consideration of the entire CPS as a ‘system of systems’ with respect to security has been neglected to date. Specifically for building automation, as a subset of CPS, no literature was discovered. Considering the totality of BASs, their many different trades and their ever-increasing interconnectivity, the question arises as to what new vulnerabilities, which have not yet been investigated in the literature, could result from the combination of individual vulnerabilities. It is recommended that future research place greater emphasis on real-world scenarios, with a view to enhancing the robustness and reliability of behavioral models. In the context of hybrid threats, particular attention must be directed towards unprotected sensor technology.

## Figures and Tables

**Table 1:** Overview and categorization of the extracted literature.

	Categories					
	Real-World Scenarios	Adoption of Standardized Vulnerability Databases	Fieldbus Systems, Protocols and Standards	Composite Vulnerabilities	Hybrid and Asymmetric Threats and Weaknesses	Intrusion Detection Systems
**Area**	CPS, ICS, IoT	BAS, CPS, ICT, IoT	BAS	CPS, ICT, IoT	CPS, ICT, IoT	CPS, ICT, IoT
**References**	[31,39,40]	[41,42,43,44,45]	[13,21,42,46]	[18,19,20,29,35,47,48,49,50,51,52,53,54,55,56,57,58,59,60]	[49,50,61,62,63,64,65,66,67,68,69,70]	[22,31,71,72,73,74,75]
**Type of Attacks or Vulnerabilities most frequently listed**	Malicious code, tampering, eavesdropping, sniffing, noise in data, gateway attacks, unauthorized access, injecting fake information, denial of service.	CVSS and NVD as very comprehensive databases that cover almost all types of attacks or vulnerabilities.	Unauthorized device discovery, denial of service, writing failure, write property, man-in-the-middle, eavesdropping, replay, spoofing, local manipulation, physical attack.	Mutual influence, inter-rule or interaction conflicts, combined vulnerabilities, man-in-the-middle, spoofing, information disclosure.	All known types of attacks or vulnerabilities. Typically divided into the cyber, physical and organizational area, physical/environ-mental damage.	Most of the known types of attacks or vulnerabilities.
**Layers affected**	Enterprise Management Automation	Enterprise Management	Field	Enterprise Management Automation Field	Enterprise Management Automation Field	Enterprise Management Automation
**Key messages**	Missing threat classification. Lack of practical examples for training deep learning algorithms and intrusion detection systems.	Focus on the field of cyber security (IT, networking). Difficult to adapt for BAS. Lack of trained personnel in the CPS area.	Due to the longevity of BAS, many old and proprietary protocols still in use. Low prevalence of BACnet Secure, KNX Secure, or other secure protocols.	Vulnerabilities that emerge from a combination of multiple components or systems, which may individually seem harmless but together lead to exploitable conditions.	Neither purely cyber, physical, or organizational nature making them challenging to detect and mitigate. Particularly in BAS, many smart sensors are located in unprotected areas, which facilitates such attacks.	IDS supports the detection of many types of threats or intrusion. Widespread use in network technology and IT. No literature available on building automation.

**Table 2:** Bus-systems and protocols with auto-discovery functionality.

Standard-, Bus-, Protocol-Name	Full Name or Short Description	Trade Mostly Spread	Pro-Prietary or Open System	Owner or Developer	Security per Design or as a Feature Implemented	Type of Security If Applicable	Object Dis-covery Tool Available	Standards Involved/Owner Link	Long Description
BACnet	Building Automation and Control	HVAC	open	bacnet.org	no		yes	ISO 16484-5; IEEE 802.2; IEEE 802.3; EIA-485, ASHRAE/ANSI 135	Communication protocol standard, object oriented, de facto standard in BAS
C-Bus	2-wire EIA-485 based	HVAC	proprietary	Honeywell	no		yes	honeywell.com	2-wire fieldbus to connect controllers amongst each other and to a BMS, only for Honeywell devices, outdated
DALI	Digital Addressable Lighting Interface	lighting	open	IEC and DiiA	no		yes	IEC 62386, IEC 60929	Widely spread lighting control bus
KNX	Konnex, formerly called EIB (Europäischer InstallationsBus) or InstaBus	lighting, electrical, HVAC	open	knx.org	no		yes	EN 50090-3,4; EN 13321-1,2; ISO/IEC 14543	Fieldbus and standard especially for lighting, shading and electrical installations, de facto standard in building automation
LON	Local Operating Network	HVAC, lighting, security	open	Echelon	no		yes	EN ISO/IEC 14908; ANSI/CEA-709.1-B	Framework: LonTalk, LonWorks, CEA-709; more outdated, very commonly used before BACnet
M-Bus	Also called Meter-Bus	metering	open	oms-group.org	no		yes	EN13757; EN 61334-4-1; IEC 60870-5	Most common bus for metering applications in BAS in Europe
M-Bus wireless	Meter-Bus as a wireless application	metering	open	oms-group.org	no		yes	EN13757-4: 2005	Uses frequency of 868MHz, designed primarily for remote reading, battery-supplied devices
Modbus RTU/ASCII	2-wire EIA-485 based	BMS, industrial	open	Modbus Organization	no		yes	modbus.org ANSI/TIA/EIA-485-A-98	Communication protocol, de facto standard for basic communication between industrial devices, royalty free
Modbus TCP/UDP	IP layer for Modbus	BMS, industrial	open	Modbus Organization	no		yes	modbus.org	Communication protocol, de facto standard for basic communication between industrial devices, royalty free
MQTT	Message Queuing Telemetry Transport	IoT, smart home	open	OASIS	yes	TLS	yes	OASIS, ISO/IEC 20922:2016	Lightweight message transport protocol for client/server environments
ONVIF	Open Network Video Interface Forum	VMS	open	onvif.org	(yes)	(TLS)	yes	onvif.org	Open industry forum that provides and promotes standardized interfaces Open industry forum that provides and promotes standardized interfaces for effective interoperability of IP-based physical security products
OPC	Open Platform Communications / OLE for Process Control	BMS	open	OPC Foundation	no		yes	opcfoundation.org	Specifies communication of real-time plant data between control devices from different manufacturers. Series of standards and specifications, based on the OLE, COM, and DCOM
OPC DA	Open Platform Communications Data Access	BMS	open	OPC Foundation	(yes)	tunneling, COM/DCOM	yes	opcfoundation.org	Client/Server communication, cross-platform, binary protocol, and web service. Intended for Alarm&Event (A&E) and History Data Access (HDA)
OPC UA	Open Platform Communications Unified Architecture	BMS	open	OPC Foundation	yes	tunneling, COM/DCOM	yes	opcfoundation.org	Client/Server communication, SOA (Service-oriented architecture), cross-platform, binary protocol and web service. Intended for Alarm&Event (A&E) and History Data Access (HDA). Unified Architecture
SMI	Standard Motor Interface	shading	open	SMI-group	no		yes	standard-motor-interface.com	5-wire common interface for sunblinds

**Table 3:** Literature in which behavioral models and BASs are mentioned in the same context.

Authors	Year	Scope	Focus Area	BAS Mentioned	Weaknesses Mentioned in the Context of Vulnerabilities	Vulnerabilities or Threat Classification	CPS/ICS System Behavior Modelling	Short Description of the Content
[82]	2017	security assessment/analysis	attack tree analysis	yes	yes	no	yes	Based on an attack tree analysis using the Markov model, the report intends to assess the BAS’s security.
[83]	2016	security assessment/analysis	BAS in general	yes	no	no	yes	Apply FTA, HAZOP, RBD, and IMECA to BAS.
[84]	2018	anomaly detection	unsupervised learning algorithm	yes	no	no	yes	Intrusion and anomaly detection via a single board computer which inspects the network traffic between the BAS nodes.

## Data Availability

The original contributions presented in this study are included in the article. Further inquiries can be directed to the corresponding author(s).

## Abbreviations

The following abbreviations are used in this manuscript:

ACS	Access Control System
AHU	Air Handling Unit
AI	Artificial Intelligence
ASHRAE	American Society of Heating, Refrigerating, and Air-Conditioning Engineers
BAS	Building Automation System
CCTV	Closed-Circuit Television
CPNI	Centre for Protection of National Infrastructure
CPS	Cyber Physical System
CVSS	Common Vulnerability Scoring System
DCS	Distributed Control System
DDC	Direct Digital Control
DOS	Denial of Service
DREAD	Damage, Reproducibility, Exploitability, Affected Users, Discoverability
EPBD	Energy Performance of Buildings Directive
FTA	Fault Tree Analysis
GPDR	General Data Protection Regulation
HAZOP	Hazard and Operability Analysis
HVAC	Heating Ventilation and Air Conditioning
IBs	Intelligent Buildings
ICS	Industrial Control System
ICT	Information and Communication Technology
IDS	Intrusion Detection System
IIoT	Industrial Internet of Things
IMECA	Intervention Mode Effects and Criticality Analysis
IoT	Internet of Things
IT	Information Technology
LAN	Local Area Network
LLMs	Large Language Models
NATO	North Atlantic Treaty Organization
NIST	National Institute of Standards and Technology
NVD	National Vulnerability Database
ODBC	Open Database Connectivity
OT	Operational Technology
RBD	Reliability Block Diagram
SBs	Smart Buildings
SCADA	Supervisory Control and Data Acquisition
SQL	Structured Query Language
STRIDE	Spoofing, Tampering, Repudiation, Information Disclosure, Denial of Service, Elevation of Privilege
TCP/IP	Transmission Control Protocol/Internet Protocol
TMM	Threat Modelling Method
VMS	Video Management System
